# Patient safety culture and incidents recorded during nursing shift changes in intensive care units

**DOI:** 10.5935/0103-507X.20220446-en

**Published:** 2022

**Authors:** Elaine Machado Oliveira, Rafaela Andolhe, Kátia Grillo Padilha

**Affiliations:** 1 Escola de Enfermagem, Universidade de São Paulo - São Paulo (SP), Brazil.; 2 Universidade Federal de Santa Maria - Santa Maria (RS), Brazil.

**Keywords:** Patient safety, Communication, Organizational culture, Perception, Safety management, Nursing assistants, Intensive care units

## Abstract

**Objective:**

To analyze the association of patient safety culture perceived by nursing professionals with incidents recorded during nursing shifts in intensive care units.

**Methods:**

This was a cross-sectional study that investigated patient safety culture measured by the Hospital Survey on Patient Safety Culture instrument. Descriptive statistics, chi-square tests, Student’s *t*-test and multiple linear regression models were analyzed considering a significance level of 5%.

**Results:**

The study reported a mean of 3.1 (standard deviation of 0.4) for the culture of patient safety in the perception of nursing professionals and 480 incidents with and without damage recorded during the nursing shifts. The variables patient safety culture with a difference between means of 0.543 (95%CI 0.022 - 1.065; p < 0.05) and nursing assistants with a difference between means of -0.133 (95%CI -0.192 - -0.074; p < 0.05) were associated with the incidents recorded during the nursing shifts. Further, nursing assistants had a lower tendency to record incidents than did the nurses.

**Conclusion:**

The strengthening of the patient safety culture and the aspects tangential to the nursing professionals represent a possible target for interventions to encourage the recording of incidents during the nursing shift shifts and improve patient safety.

## INTRODUCTION

The patient safety culture from the perspective of nursing professionals is an important aspect of nursing practices for the outcomes of patients in intensive care.^(1-6)^ An important component of the practice is shift changes, which encompass concepts of the patient safety culture, including the ability to communicate among professionals, leadership, recording and notifying others of incidents, and these practices imply the positioning of nurses to make appropriate decisions.^(1,2,4,6,7)^ Although sometimes neglected, shift changes integrate aspects of patient safety culture that are fundamental to the continuity of care and safety.

Patient safety culture on nursing professionals’ perception can be measured through instruments that identify how and when nurses can intervene and develop strategies for patient safety. A widely used instrument is the Hospital Survey on Patient Safety Culture (HSOPSC),^(7)^ created by the Agency for Research and Quality in Health Care (AHRQ), translated and validated for Brazilian culture.^(6)^

Currently, the literature presents many studies that have applied the HSOPSC to evaluate health professionals’ perceptions, including nurses, regarding patient safety culture.^(1-3,5,8,9)^ These studies identify a low percentage of positive responses, ranging between 8.0% and 59.7%^(1-3,5,8,9)^ for the dimension of communication openness; between 1.6% and 43.3%^(1,2,5,9)^ for nonpunitive response to errors; between 4.8% and 55.8%^(1-3,5,8,9)^ for the frequency of event reporting; between 4.8% and 52.8%^(1-3,5,8,9)^ for hospital hand-offs and transitions; and 1.6%,^(1)^ 23.4%,^(3)^ 36.3%,^(9)^ 52.8%^(2)^ for feedback and communication about error.

However, other important results with a greater proportion of positive responses, according to the nurses, show that in the units with 66.9%^(5)^ positive responses, the dimensions of teamwork that contribute most to patient safety are supervisor/manager expectations and actions promoting safety (65.8%^(5)^ and 74.3, respectively),^(2)^ organizational learning/continuous improvement (69.2%)^(3)^ and staffing (65.7%).^(2)^ These results represent strengthened aspects of the patient safety culture that can be used to support improved nursing practices and patient safety.

Therefore, considering the results already published, nursing shift changes, rarely investigated in the literature, are crucial for the continuity of care and can contribute to recording and notifying incidents, mainly with the possibility of intervening in situations that can prevent new incidents.

This study highlights the importance of investigating the patient safety culture on nursing professionals’ perception and identifying incidents recorded during nursing shift changes to intervene with best nursing practices and ensure continuity of care. As a hypothesis, the study proposes that the patient safety culture on nursing professionals’ perception is associated with the incidents recorded during nursing shift changes.

To this end, the objective is to analyze the association of the patient safety culture on nursing professionals’ perception with the incidents recorded during nursing shift changes in intensive care units (ICUs).

## METHODS

A cross-sectional study was conducted in 8 ICUs of a university hospital with a capacity for approximately 1,000 beds, with 74 ICU beds distributed among clinical emergency ICU (12 beds), surgical emergency (16 beds), medical clinic (8 beds), infectious disease (7 beds), general surgery (11 beds), neurology (11 beds), nephrology (5 beds) and burn units (4 beds).

In 2015, the institution created a Risk Management Group to implement programs to report adverse events and work on patient safety. The group was composed of nurses who worked in partnership with the nursing board in the management of all incidents occurring during nursing practices in the institution.^(10)^

In ICUs, nurses and nursing assistants have different duties. Nurses are responsible for invasive interventions and specialized care, such as hemodynamic monitoring, transport of critically ill patients, and staff supervision, among others. Nursing assistants have a level of technical training and are responsible for noninvasive care, such as recording vital signs, mobilization, hygiene, nonvasoactive medications, and enteral nutrition.

To identify the nursing shift changes to be monitored, a randomized random sampling was performed. Through permuted randomization, a sampling of 10% of all nursing shift changes performed in each ICU during the study period was calculated. Considering unit, date, and time, there were 390 total follow-ups.

For the convenience sample of nursing professionals, all nurses and nursing assistants who worked in any of the eight ICUs during the study period were included, regardless of the working time in the units.

The dependent variable of the study was represented by the incidents recorded during the nursing shift changes. The independent variables of the nursing professionals included in the regression model were professional category (nurses and nursing assistants), age, sex (male and female), working time in the ICU, job satisfaction (yes or no), and patient safety culture on nursing professionals’ perception reported on the HSOPSC.

The HSOPSC instrument consists of 12 dimensions, with seven at the level of care (supervisor/manager expectations and actions promoting safety, organizational learning/continuous improvement, communication openness, feedback and communication about error, teamwork within unit, staffing and nonpunitive response to errors); three at the organizational level (teamwork across hospital units, hospital hand-offs and transitions, and hospital management support for patient safety); and two at the level of individual results (overall perceptions of patient safety and frequency of event reporting).^(6)^

The 12 dimensions consist of 42 items with responses measured on a 5-point Likert scale ranging from 1 point for total disagreement to 5 points for total agreement. The results are measured as a percentage of positive responses and indicate a strong culture when 75% or more of the responses are positive and a weak culture when only 50% or less are positive. ^(6)^ In this study, the patient safety culture was also analyzed using the mean of positive responses with results ranging from 1.0 to 5.0, indicating a strengthened safety culture when the means were above 2.5 and a weakened safety culture when the means were below 2.5.

The instrument also presents the evaluation of nurses on the outcome variables of patient safety levels and reporting events in the previous 12 months. ^(6)^ This last variable was not included in this study because the incidents were recorded *in loco* during nursing shift changes.

Two researchers collected the completed data instruments following ethical principles. The professionals who agreed to participate in the study received the instruments in sealed envelopes and completed them in an average time of 1 hour. All questions about the instruments were clarified to ensure full completion.

For the incidents, 12 trained monitors (undergraduate and graduate nursing students) monitored the nursing shift changes and recorded all incidents reported by each nursing professional.

The incidents recorded by the monitors were analyzed by two researchers using double data checking. Doubly recorded incidents were considered only once. Subsequently, the manual of the International Classification for Patient Safety (ICPS) of the World Health Organization (WHO) was applied to classify the incidents according to the severity of the damage: severe, moderate, mild, or incidents without damage.^(11)^ Next, the researchers analyzed the patient’s medical records to identify whether the incidents recorded during nursing shift changes had been reported in the patient’s medical records.

All data were entered into Excel and, after double-checking, imported and processed with the Statistical Package for the Social Sciences (SPSS), version 19.0. Descriptive statistics were performed with absolute and relative frequencies, in addition to measures of central tendency (mean and standard deviation - SD).

The comparative analysis of qualitative variables among nursing professionals was performed using the chi-square test. The normality and homogeneity of the quantitative variables were analyzed using the Kolmogorov‒Smirnov test and the Levene test, respectively. Student’s *t* test was applied to compare the groups of nurses and nursing assistants. The association of patient safety culture and the other variables of nursing professionals with the incidents recorded during nursing shift changes was performed using the multiple linear regression model.

The internal consistency of the HSOPSC instrument for safety culture was analyzed using Cronbach’s alpha coefficient. This measure was consistent with a result greater than 0.712. All tests were performed considering a significance level of 5%.

The study was approved by the Ethics Committee of the institution under protocol 196/11 and conducted according to the ethical standards required by resolution 466 of December 12, 2012. After clarifying the objectives of this study and its benefits, as well as ensuring the anonymity of the participants, the Free and Informed Consent Form was signed in duplicate, and one copy remained with the researchers.

## RESULTS

Among the 287 (83.4%) nursing professionals who agreed to participate in the study, 100 (34.8%) were nurses, and 187 were nursing assistants (65.2%). The sample consisted mostly of females, with 241 (84.0%) women; 257 were satisfied with their work in the ICU (89.5%), with a statistically significant difference for both variables (p < 0.05).

The nurses had a mean age of 37.5 years (SD = 8.3), and the mean age of nursing assistants was 39.4 years (SD = 8.9). There was no statistically significant difference in age (p = 0.39). The mean length of work in the ICU was 7.8 years (SD = 5.2) for nurses and 9.2 years (SD = 6.1) for nursing assistants, with a statistically significant difference between the groups (p < 0.05). The patient safety culture on nurses’ perception was 3.05 (SD = 0.10) and on nursing assistants’ perception was 3.06 (SD = 0.11), with no statistically significant difference between categories (p = 0.38) as shown in table 1.

**Table 1:** Descriptive measures of nursing professionals

Variables	Nurses	Nursing assistants	p value^*^
Age	37.5 (8.3)	39.4 (8.9)	0.39
Length of ICU work	7.8 (5.2)	9.2 (6.1)	< 0.05
Patient safety culture	3.05 (0.10)	3.06 (0.11)	0.38

ICU - intensive care unit. Results expressed as the mean (standard deviation).

* Student’s *t* test.

For the analysis of patient safety culture, the proportion of positive responses on the HSOPSC dimensions and the means are shown in table 2.

**Table 2:** Proportion and descriptive measure of positive responses to the patient safety culture by dimension of the Hospital Survey on Patient Safety Culture

Dimensions	n (%)	Mean (SD)
Supervisor/manager expectations and actions promoting safety	190 (66.2)	3.6 (0.7)
Organizational learning/continuous improvement	165 (57.5)	3.3 (0.7)
Communication openness	120 (41.8)	3.2 (0.8)
Feedback and communication about error	99 (34.4)	3.1 (0.9)
Teamwork within unit	137 (47.7)	2.9 (0.7)
Staffing	65 (22.6)	2.4 (0.7)
Nonpunitive response to errors	61 (21.2)	2.3 (0.7)
Teamwork across hospital units	137 (47.7)	3.1 (0.6)
Hospital hand-offs and transitions	150 (52.2)	3.0 (0.7)
Hospital management support for patient safety	130 (45.3)	3.0 (0.8)
Overall perceptions of patient safety	129 (44.9)	3.6 (0.6)
Frequency of event reporting	170 (59.2)	3.6 (1.0)

SD - standard deviation. Culture strengthened to a proportion of positive responses above 75% and fragile to 50% or less.^(6)^ Strong safety culture for means above 2.5 and fragile for means below 2.5.

Table 2 shows a higher proportion of positive responses to the dimension supervisor/manager expectations and actions promoting safety (66.2%). The dimensions of frequency of event reporting (59.2%), organizational learning/continuous improvement (57.5%), hospital hand-offs and transitions (52.2%) showed a proportion of positive responses above 50%. The dimensions that presented a proportion below 50% were nonpunitive response to errors (21.2%), staffing (22.6%), and feedback and communication about error (34.4%).

Most professionals (54.9%) considered the patient safety culture in ICUs to be acceptable, 35.9% considered it very good or excellent, and 9.2% considered the patient safety culture to be weak or flawed.

The mean patient safety culture for all nursing professionals was 3.1 (SD = 0.4). The dimensions with the highest means indicating a strengthened safety culture on nursing professionals’ perception were supervisor/manager expectations and actions promoting safety (3.6; SD = 0.7), overall perceptions of patient safety (3.6; SD = 0.6), and frequency of event reporting (3.6; SD = 1.0). The dimensions that obtained the lowest means were nonpunitive response to errors (2.3; SD = 0.7), staffing (2.4; SD = 0.7), and teamwork within unit (2.9; SD = 0.7).

During the monitoring of the 390 nursing shift changes, 480 damaging and nondamaging incidents were recorded. However, after analyzing the patient’s medical records, it was found that of the total number of recorded incidents, less than half were reported in the medical records (n = 203; 42.3%), as shown in table 3.

**Table 3:** Incidents recorded during nursing shift changes, considering the severity of the damage and the incidents reported in the patients’ medical records

Incidents	Severe	Moderate	Light	No damage	Total
Recorded during nursing shift changes	114 (23.7)	224 (46.7)	95 (19.8)	47 (9.8)	480 (100.0)
Recorded in patient’s medical records	45 (39.5)	108 (48.2)	38 (40.0)	12 (25.5)	203 (42.3)

Results expressed as n (%).

According to the degree of damage, incidents recorded during nursing shift changes had a higher proportion of moderate injury (46.7%), followed by incidents with severe (23.7%) and mild (19.8%) injury. Incidents without damage accounted for 9.8%.

The association of patient safety culture and the variables of nursing professionals with the incidents recorded during nursing shift changes is shown in table 4.

**Table 4:** Association of patient safety culture with the incidents recorded during nursing shift changes

Incidents	Difference between means	95%CI	p value^*^
Professional category of nursing assistants	-0.133	-0.192 - -0.074	< 0.05
Female gender	-0.070	-0.147 - 0.007	0.073
Satisfied at work	0.034	-0.153 - 0.222	0.718
Age	0.000	-0.008 - 0.008	0.948
Length of ICU work	-0.003	-0.015 - 0.009	0.590
Patient safety culture	0.543	0.022 - 1.065	< 0.05

95%CI - 95% confidence interval. ICU - intensive care unit.

* Multiple linear regression model.

Table 4 shows that the variables patient safety culture and the category of nursing assistants showed a significant association with the incidents recorded during nursing shift changes. These variables were significant predictors for the incidents recorded during nursing shift changes. The patient safety culture on nursing professionals’ perception was associated with an average increase of 0.543 points (95% confidence interval - 95%CI 0.022 - 1.065; p < 0.05) in the mean of the recorded incidents. The category of nursing assistants was associated with a lower identification of incidents recorded during nursing shift changes than the category of nurses, with an average of -0.133 points (95%CI -0.192 to -0.074; p < 0.05).

## DISCUSSION

The study identifies that patient safety culture is significantly associated with incidents recorded during ICU nursing shift changes, with a difference between the means of 0.543 (95%CI 0.022 - 1.065; p < 0.05), and the category of nursing assistants is significantly associated with a lower number of recorded incidents than the category of nurses, with a difference between the means of -0.133 (95%CI -0.192 - -0.074; p < 0.05).

Regarding patient safety culture on nursing professionals’ perception, the proportion of positive responses was below 75% for each dimension of the instrument. In addition, 54.9% of the ICU professionals in the study reported only an acceptable patient safety culture, indicating fragility for the professionals in this study. Comparing the two categories, the mean of patient safety culture was similar between nursing assistants (3.06; SD = 0.11) and nurses (3.05; SD = 0.10), and the difference between the groups was not statistically significant (p = 0.38). The study shows a low trend of recording incidents in the patient’s medical records. Less than half (42.3%) of the total number of incidents recorded during nursing shift changes was reported in the patient’s medical records.

These results represent very important aspects of nursing practices to be explored by the institution and by nurses to implement interventions to increase the recording and notification of incidents and to promote patient safety. The investment in factors constituting a patient safety culture, especially those with higher proportions of positive responses, may favor the recording of incidents during nursing shift changes. Furthermore, considering that the incidents were identified by monitors and not by the professionals themselves, the study suggests that the presence of nursing assistants favors the recording of incidents; however, the negative coefficient indicates that they tend to record less than nurses. This result validates the fundamental presence of nurses in ICUs, either as professionals who record the incident or as a positive influence on nursing assistants, for incidents recorded during nursing shift changes.

Studies in the literature reinforce these findings by identifying that investment in nursing processes and the participation of nurses in the ICU promote communication, the feeling of safety in decision-making, and the empowerment of professionals to report incidents, which directly favors patient safety, especially in treating and preventing new incidents.^(2,3,8)^

Another study also confirms that nurses make a direct contribution to the patient safety culture. The results show that nurses have a higher proportion of positive responses than physicians, physiotherapists, and nursing assistants in the dimensions related to communication, such as feedback and communication about error, communication openness, nonpunitive response to errors, and hospital hand-offs and transitions.^(9)^

Therefore, the literature reinforces that it is crucial to effectively include nurses in the organizational processes of ICUs and motivate the participatory leadership, training and support of managers who can take measures to promote a patient safety culture and prevent incidents.^(2,3,5)^

Among the 12 dimensions of the HSOPSC, supervisor/manager expectations and actions promoting safety, frequency of event reporting, organizational learning/continuous improvement, and hospital hand-offs and transitions show proportions of positive responses above 50.0% and averages above 2.5. Although they are not fragile, the proportions are very close to 50.0%, and the means close to 2.5 reinforce the need to invest in and strengthen the patient safety culture in ICUs. International studies that analyzed the patient safety culture on nurses’ perception also found higher proportions of positive responses for dimensions related to organization and management.^(2,3,5,8)^

The dimensions of communication openness and feedback and communication about error show the weaknesses in communication in the units of this study. Regarding the dimensions related to communication, the literature also shows proportions of positive responses below 50%^(1,2,5,8,9)^ suggesting weakness of the patient safety culture in the aspects of communication and indicating only acceptable or weak patient safety cultures for nurses and nursing assistants.^(8^.^9)^

These results reinforce the challenge of investing in ICU patient safety culture to promote better patient outcomes. A study that analyzed the recommendations of nursing professionals to strengthen the patient safety culture suggested teamwork is a key aspect in improving the communication of incidents and safety culture.^(5)^

Other important aspects for strengthening the patient safety culture identified in this study and reinforced by the literature^(2,3,5)^ as a possibility for interventions include the organizational and management aspects widely studied in the institution since 2015 when the Risk Management Group was developed to invest in programs for the notification of adverse events, implementation of electronic patient records, protocols and specific instruments for the management of nursing practices.^(10)^

Regarding the incidents investigated in this study, more incidents were recorded during nursing shift changes due to pressure injury, lack of documentation, and pulmonary infection. Other studies investigating the occurrence of incidents also found a higher proportion of pressure injuries.^(12,13)^

Regarding the severity of the damage, this study found higher proportions of incidents with moderate to severe damage, with only 19.8% of incidents having light damage and 9.8% having no damage. These results may indicate the concern of nursing professionals in reporting serious and moderate incidents to ensure the continuity of care.

A Brazilian study that investigated incidents involving elderly patients recorded during nursing shift changes in the ICU found that 29.8% of them suffered 183 incidents with moderate to severe damage, with an average of 1.9 incidents per patient and a higher proportion related to clinical procedures.^(13)^

Finally, addressing the hypothesis of the study, the patient safety culture on nursing professionals’ perception, besides the category of nursing assistants, is associated with the incidents recorded during nursing shift changes in ICUs. This result indicates that by promoting the aspects that make up the patient safety culture in ICUs, especially in the dimensions with higher proportions of positive responses, it is possible to promote the recording of incidents during nursing shift changes. In addition, the presence of nursing assistants and nurses in ICUs also promotes the recording of incidents.

This study has limitations that include the sample being drawn from a single institution, making it difficult to generalize the results to other institutions with differences in human resources and organizational structure and processes. Thus, the findings presented in this study may not be representative of contexts other than that in which it was performed.

Another limitation presented by this study includes its design and the reverse causality bias that does not allow for establishing causality between the variables analyzed. An additional limitation is a lack of including organizational variables that may interfere with the notification of incidents, such as the inclusion of nurse managers in the sample, the evaluation of leadership and management tools, and the notification of incidents in the institution.

## CONCLUSION

The patient safety culture on nursing professionals’ perception in intensive care units and the category of nursing assistants are factors associated with incidents recorded during nursing shift changes. The study also shows that nursing assistants have a lower tendency to record these incidents than nurses. Thus, the investment in patient safety culture and aspects tangential to nursing assistants and nurses represents possible targets for intervention to encourage the recording of incidents during nursing shift changes and improve patient safety in intensive care units.
